# High-risk and low-risk human papillomavirus detection in self-collected samples compared to clinician-collected samples in a Polish population

**DOI:** 10.3389/fpubh.2026.1804719

**Published:** 2026-04-21

**Authors:** Sandra Góral, Anne Liljander, Marco Kai, Markus Cavalar, Melanie Harder

**Affiliations:** 1 Genetic Department, Euroimmun Polska sp. z o.o., Wroclaw, Poland; 2 Institute of Experimental Immunology, Affiliated to EUROIMMUN Medizinische Labordiagnostika AG, Lübeck, Germany

**Keywords:** cervical cancer, epidemiology, human papillomavirus, Poland, self-sampling, women's health

## Abstract

**Introduction:**

Persistent infections with human papillomavirus (HPV) cause approximately 95% of cervical cancers. The introduction of cytology screening has, however, significantly reduced morbidity and mortality rates. More recently, HPV DNA testing has been shown to be more sensitive than traditional cytology-based testing in detecting cervical intraepithelial neoplasia (CIN) grade 2 or worse (CIN2+). Self-sampling for HPV testing offers a viable alternative to clinician-performed sampling and could potentially increase participation rates in organized screening programs. Here, we compared the accuracy of self-collected vaginal samples with clinical-collected cervical samples for HPV-based screening and evaluated the acceptability of HPV self-sampling among Polish women.

**Materials and methods:**

Women with unclear or abnormal results at their last cytology screening were invited to participate. Each participant submitted two swabs for HPV testing: one clinical-collected cervical swab and one self-collected vaginal swab (Evalyn^®^ Brush). Both sample types were subsequently tested with the EUROArray HPV assay. Participants also completed a questionnaire to assess their experiences with the self-sampling procedure and express their opinions on it.

**Results:**

In total, 180 women aged 30 to 70 years were recruited. The prevalence of high-risk (hr) HPV was 56.7% in clinician-collected samples and 55.0% in self-collected samples. Overall concordance for any HPV, hr-HPV and low-risk (lr) HPV detection between self-collected and clinician-collected samples was 88.9%, 86.1%, and 85.0%, respectively. Most participants found self-sampling comfortable (82.4%), useful (87.0%), and convenient (85.8%) and felt low/very low stress (58.0%) while sampling. In total, 81.3% of the women reported that it was practically painless and 77.2% felt less embarrassed. Of the respondents, 51.1% preferred clinician-collected sampling, 40.9% preferred self-sampling, while 4.6% had no preference.

**Conclusions:**

Vaginal self-sampling using the Evalyn^®^ Brush showed substantial agreement with cervical clinician-sampling for HPV detection using the EUROArray HPV test. Self-sampling for HPV testing was well-accepted by the participants. It represents a feasible alternative for clinical sampling in Poland, which could be effectively integrated into the national HPV screening program.

## 1 Introduction

Cervical cancer is the fourth most common cancer in women worldwide, with an estimated 662,301 new cases and 348,874 deaths in 2022 (1). Persistent infections with human papillomavirus (HPV) cause approximately 95% of cervical cancers, with high-risk (hr) HPV subtypes 16 and 18 being responsible for approximately 70% of cases (2). Other co-factors such as low socioeconomic status, smoking, multiple sexual partners, repeated childbirths, use of oral contraceptives, and a history of sexually transmitted infections increase the risk of cervical cancer development (3).

Incidence and mortality rates of cervical cancer have decreased following the implementation of organized cytology-based screening programs. Today, most cancer cases occur in countries lacking high-quality screening programs, or among women who do not participate in existing programs. According to previous studies, several social, economic, and cultural factors contribute to non-attendance, with the most common reasons being lack of time, fear of screening, embarrassment and discomfort associated with the gynecologic examination (4–6).

HPV DNA testing has been shown to be more sensitive than traditional cytology-based testing for detecting cervical intraepithelial neoplasia (CIN) grade 2 or worse (CIN2+) (7), providing 60%−70% higher protection against invasive cervical carcinoma (8). Consequently, HPV testing of clinician-collected cervical samples is now replacing traditional cytology as the primary screening method in many programs (9). Furthermore, HPV testing can be performed on self-collected vaginal samples, showing comparable diagnostic accuracy for detecting CIN2+ to clinician-collected samples (10). This self-sampling approach has been introduced in several countries to improve screening coverage, particularly among women not attending regular screenings (11).

In Poland, a nation-wide cytology-based screening program was introduced in 2006/2007, offering women aged 25–64 years testing every 3 years (12, 13). Data suggests that implementation of this program has accelerated declines in both cervical cancer incidence and mortality (14). However, the number of cases and related deaths remain higher in Poland than in many Western European countries (15). The low participation rate in the publicly funded screening program (11.3% in 2023), fragmented access to gynecological care and suboptimal cytology performance may contribute to this gap (16–19).

In 2025, the nation-wide screening cervical cancer prevention program was changed. The new scheme involves performing an hr-HPV test with genotyping, but data regarding participation and quality of the services is limited (50).

Offering vaginal self-sampling for HPV testing could increase participation and detection rates. However, studies evaluating both the diagnostic accuracy of self-sampling and its acceptability among Polish women are limited. Therefore, the aim of this study was to assess whether HPV screening of self-collected vaginal samples is as accurate as that of clinician-collected cervical samples, and whether HPV self-sampling is acceptable to Polish women.

## 2 Material and methods

### 2.1 Study population

Women aged 30 years and older, attending participating clinics in Lower Silesia, Poland (CORFAMED Women's Health Center Sp. z o.o., Wroclaw, Lower Silesian Oncology Center, Wroclaw, “Healthy Woman” Medical Center Sp. z o.o., Wroclaw, MARIMAR Mariusz Markuszewski running medical company the Lower Silesian Gynecology Center, Wroclaw and Specialist Medical Practice Mateusz Tylko, Wroclaw) were invited to participate face-to-face during clinic visits. Eligibility required participants to be ≥30 years and to have an unclear or abnormal cytology result (≥ASC-US) at their last screening. Written informed consent was obtained from all participants. This study was conducted between December 2015 and November 2018. The Bioethics commission at the Medical University in Wroclaw approved the study (No. KB – 170/2016).

### 2.2 Specimen collection and self-sampling kit

During gynecological examination, a cervical swab sample was collected by a clinician using a dry cone-shaped flocked swab (FLOQSwabs^®^, Copan Italia, Italy). Swabs were stored dry and shipped at ambient temperature to EUROIMMUN DNA Genetic Laboratory (Wroclaw, Poland) for subsequent analysis. After the examination, participants received a self-sampling kit for home collection, including an Evalyn^®^ Brush (Rovers Medical Devices B.V., Oss, Netherlands), a liquid absorbent pad, a pair of gloves, a safety bag, a return envelope, and detailed written and illustrated instructions. Patients were advised to collect the vaginal sample on day 4 after collection by the clinician to allow for epithelial recovery. Samples were returned by courier along with the signed consent form and questionnaire.

### 2.3 DNA extraction and HPV genotyping using the EUROArray HPV

DNA extraction was performed at the EUROIMMUN DNA Genetic Laboratory using the QIAamp^®^ DNA Mini Kit (Qiagen, Hilden, Germany) and a modified spin protocol. Briefly, dry swabs were placed in 400 μl sterile phosphate-buffered saline (PBS), mixed by vortexing and briefly centrifuged. The sample material was transferred into a new clean tube, followed by the successive addition of 20 μl Proteinase K and 400 μl buffer AL, followed by vortexing and brief centrifugation. The samples were incubated at 56 °C for 10 min and briefly centrifuged. In the next step, 400 μl ethanol was added. The samples were vortexed and briefly centrifuged. In total, 500 μl of the obtained mixture was added to the QIAamp Mini spin column and centrifuged (8,000 rpm, 1 min). The final step was repeated with the remaining mixture. Then, 500 μl Buffer AW1 was added to the QIAamp Mini spin column and centrifuged (8,000 rpm, for 1 min). Next, 500 μl Buffer AW2 was added to the QIAamp Mini spin column and centrifuged (14,000 rpm, 3 min). The QIAamp Mini spin column was centrifuged again (at full speed, 1 min). After every centrifugation a new clean tube was used to collect the filtrate. In total 50 μl Buffer AE (heated to 56 °C) was added to the QIAamp Mini spin column, incubated at room temperature (15–25 °C) for 5 min and then centrifuged (8,000 rpm,1 min). A total of 50 μl was obtained from a single sample. The eluates were stored at 2–8 °C until subsequent analysis. Negative controls consisting of clean swabs (Z.T.S HAGMED B030, Hagmed, Rawa Mazowiecka, Poland) were included in each extraction and PCR run.

HPV typing was conducted with the EUROArray HPV test (EUROIMMUN Medizinische Labordiagnostika AG, Lubeck, Germany) according to the manufacturer's instructions. The test detects and differentiates 30 anogenital HPV genotypes−18 high-risk (hr) HPV types (16, 18, 26, 31, 33, 35, 39, 45, 51, 52, 53, 56, 58, 59, 66, 68, 73, and 82) and 12 low-risk (lr) HPV types (6, 11, 40, 42, 43, 44, 54, 61, 70, 72, 81, and 89)—by amplification of viral E6/E7 oncogenes and microarray hybridization. By using subtype specific primer and probe systems, HPV genotypes were detected and differentiated simultaneously in one multiplexed reaction. Human genomic DNA amplification served as internal control. The hybridization of the PCR product to the corresponding probe was detected using the EUROArrayScanner. The EUROArrayScan software subsequently evaluated all spot signals (relative fluorescent intensity) and generated a qualitative test result, i.e., detected/not detected based on HPV-type specific cut-offs.

### 2.4 Questionnaires

Participants assessed their experience with the self-sampling procedure using a three-part questionnaire covering:

1. Practical aspects of the self-sampling kit (readability, comfort, usability, and convenience).
2. Perceived feelings during the self-sampling (stress, self-confidence, pain, and embarrassment).
3. Preference for self- vs. clinician-collection in future screenings.

Responses were rated on a five-point Likert scale.

### 2.5 Statistical analysis

Age was categorized into three groups: 30–39, 40–49, and ≥50 years of age. Comparisons between clinician- and self-collected samples were evaluated using contingency tables. Agreement parameters included overall agreement, positive percent agreement (PPA) and negative percent agreement (NPA). Cohen's kappa (κ) coefficients with 95% confidence intervals were calculated and interpreted as: slight (0–0.20); fair (0.21– 0.40); moderate (0.41–0.60); substantial (0.61–0.80); and almost perfect (0.81–1.00) (20). The McNemar test was applied to assess differences in genotype detection.

Since the EUROArray HPV detects and differentiates 30 HPV types (18 hr-HPV and 12 lr-HPV), agreement between the two collection methods was evaluated for each genotype individually as well as pooled across groups (any HPV, hr-HPV, and lr-HPV).

For analysis of individual genotypes, paired samples were classified as:

- Concordant: identical genotype(s) or both negative.
- Partly concordant: at least one identical genotype.
- Discordant: no shared genotype(s) similarities.

Descriptive statistics were used to summarize results on women's acceptance of the self-sampling procedure. Statistical analyses were performed using R version 3.6.2 (2019-12-12) (51).

## 3 Results

### 3.1 Participants characteristics

Paired clinician-collected cervical and self-collected vaginal samples from 180 women aged 30–70 years (mean 42.0 years) were analyzed. Questionnaires were returned by 176 participants (97.8%). Only 35.6% (64/180) followed the recommended 4-day interval before self-collection. Instead, 13.9% (25/180) of the women collected their vaginal sample before day 4 and 50.6% (91/180) collected it ≥5 days after the cervical sample (range: 5–43 days). Internal control amplification was successful in all samples, confirming adequate sample collection.

### 3.2 HPV detection

HPV of any genotype was detected in 66.7% (120/180) of the clinician-collected and 65.6% (118/180) of the self-collected samples. Hr-HPV and lr-HPV prevalence were 56.7% (102/180)/55.0% (99/180) and 35.6% (64/180)/35.0% (63/180), respectively.

- overall agreement: 88.9% (95% CI: 83.4% to 93.1%), κ was 0.75.
- hr-HPV agreement: 86.1% (95% CI: 80.3% to 90.4%), κ was 0.72.
- lr-HPV agreement: 85.0% (95% CI: 79.1% to 89.5%), κ was 0.67 (Table 1).

**Table 1:** Agreement in HPV detection between 180 paired clinician-collected cervical samples and self-collected vaginal samples.

Self-collected	Clinician-collected		Overall agreement (95% CI)	PPA (95% CI)	NPA (95% CI)	Kappa (95% CI)
	**Positive (%)**	**Negative (%)**				
Any HPV						
Positive (%)	109 (57.5)	9 (6.2)	88.9 (83.4–93.1)	90.8 (84.3–94.8)	85.0 (73.9–91.9)	0.75 (0.65–0.85)
Negative (%)	11 (5.8)	51 (30.5)				
hr-HPV						
Positive (%)	88 (47.3)	11 (7.1)	86.1 (80.3–90.4)	86.3 (78.3–91.6)	85.9 (76.5–91.9)	0.72 (0.62–0.82)
Negative (%)	14 (7.1)	67 (38.5)				
lr-HPV						
Positive (%)	50 (28.3)	13 (8.0)	85.0 (79.1–89.5)	78.1 (66.6–86.5)	88.8 (81.8–93.8)	0.67 (0.56–0.79)
Negative (%)	14 (6.6)	103 (57.1)				

PPA, positive percent agreement; NPA, negative percent agreement; hr-HPV, high-risk HPV; lr-HPV, low-risk HPV; CI, confidence interval.

The prevalence of HPV was highest in women aged 30–39 years. Substantial agreement was observed across all age groups (Table 2).

**Table 2:** Prevalence and overall agreement in HPV detection between clinician-collected samples and self-collected samples, categorized by the patients' age.

Age category (years)	No. women	Clinician-collected	Self-collected	Overall agreement (95% CI)	Kappa (95% CI)
		HPV + (%)	HPV + (%)		
30–39	87	64 (73.6)	60 (69.0)	90.8 (82.7–96.0)	0.77 (0.63–0.92)
40–49	56	39 (69.6)	37 (66.1)	89.3 (78.1–96.0)	0.75 (0.57–0.94)
≥50	37	17 (46.0)	21 (56.8)	83.8 (68.0–93.8)	0.68 (0.45–0.91)
		hr-HPV + (%)	hr-HPV + (%)		
30–39	87	54 (62.1)	52 (59.8)	90.8 (82.7–96.0)	0.81 (0.68–0.93)
40–49	56	33 (58.9)	30 (53.6)	84.0 (71.7–92.4)	0.67 (0.48–0.87)
≥50	37	15 (40.5)	17 (46.0)	78.4 (61.8–90.2)	0.56 (0.29–0.83)
		lr-HPV + (%)	lr-HPV + (%)		
30–39	87	35 (40.2)	32 (36.8)	78.2 (68.0–86.3)	0.54 (0.36–0.72)
40–49	56	17 (30.4)	17 (30.4)	92.9 (82.7–98.0)	0.83 (0.67–0.99)
≥50	37	12 (32.4)	14 (37.8)	89.2 (79.1–89.5)	0.76 (0.55–0.98)

hr-HPV, high-risk HPV; lr-HPV, low-risk HPV; CI, confidence interval.

### 3.3 HPV genotyping

Of the HPV-positive cervical samples, 48.3% (58/120) showed single-type and 51.7% (62/120) multiple-type infections (range 2 to 8 types). Of the self-collected samples, 41.5% (49/118) showed single-type and 58.5% were multiple-type infections (69/118, range 2 to 10 types).

**Figure 1:**
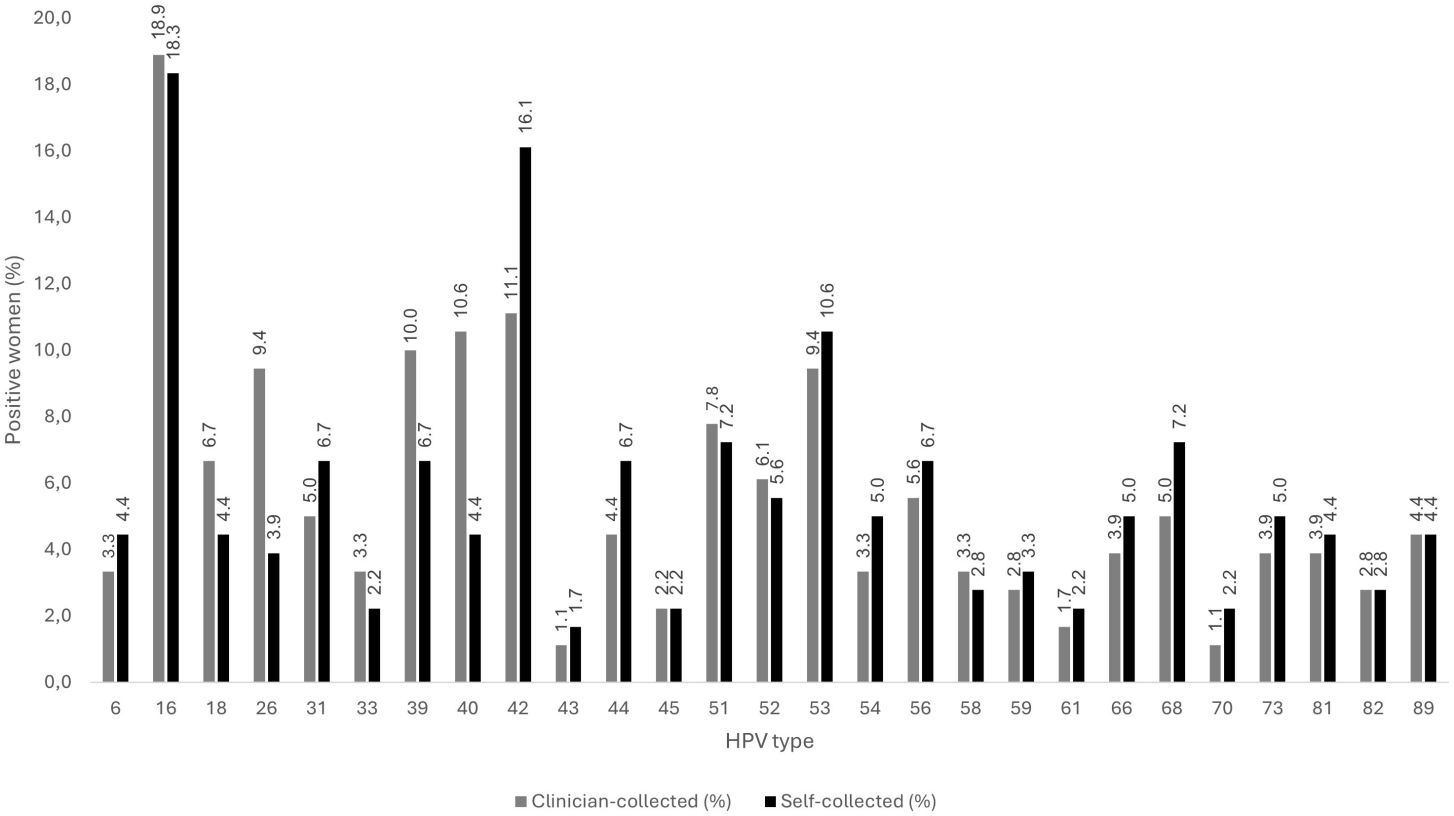
Prevalence of the different HPV types in the 180 Polish women participating in the study.

A total of 27 HPV genotypes were identified. HPV 16 was the most prevalent (clinician 18.9%, self 18.3%) followed by HPV type 18 (6.7% vs. 4.4%). The prevalence of the individual HPV genotypes in clinician- and self-collected samples is depicted in Figure 1.

The overall agreement for HPV 16 detection was 96.1% (κ = 0.87), almost perfect agreement, whereas for HPV 18 it was 94.4% (κ = 0.47, moderate agreement). The agreement for the detection of each individual HPV genotype is depicted in Table 1. Certain HPV types were more frequently detected in one sample type than in the other, for example, hr-HPV 26 (9.4% vs. 3.9%) and lr-HPV 40 (10.6% vs. 4.4%) were more common in clinician-collected samples, while lr-HPV 42 (16.1% vs. 11.1%) was more often found in self-collected samples (Figure 1). The differences were statistically significant for hr-HPV 26 (*p* = 0.037) and for lr-HPV 40 (*p* = 0.026).

### 3.4 Acceptability of self-collection

Most women rated self-sampling positively:

- Instructions readability: 95.5% positive/very positive
- Comfort, usability and convenience: 82.4%−87.0% positive/very positive
- Stress: low/very low in 58.0%
- Pain: low/very low in 81.3%
- Embarrassment: low/very low in 77.2% of the women

In total, 51.1% preferred clinician sampling, 40.9% preferred self-sampling, 4.6% had no preference (Tables 3–5).

**Table 3:** Rating of the practical aspects of the vaginal self-sampling kit among the 176 women who returned their questionnaires.

Rating of experience	Readability of manual (%)	Collection comfort (%)	Usability (%)	Convenience (%)
Very positive	86 (48.9)	67 (38.1)	76 (43.2)	82 (46.6)
Positive	82 (46.6)	78 (44.3)	77 (43.8)	69 (39.2)
Neither positive nor negative	5 (2.8)	27 (15.3)	20 (11.4)	19 (10.8)
Negative	2 (1.1)	1 (0.6)	1 (0.6)	3 (1.7)
Very negative	1 (0.6)	1 (0.6)	0 (0.0)	1 (0.6)
No answer	0 (0.5)	2 (1.1)	2 (1.1)	2 (1.1)

**Table 4:** Rating of the feelings perceived during the self-sampling process.

Rating of experience	Stress (%)	Self-confidence (%)	Pain (%)	Embarrassment (%)
Very high	9 (5.1)	13 (7.4)	1 (0.6)	2 (1.1)
High	24 (13.6)	47 (26.7)	0 (0.0)	3 (1.7)
Neither low nor high	34 (19.3)	70 (39.8)	22 (12.5)	25 (14.2)
Low	57 (32.4)	23 (13.1)	26 (14.8)	21 (11.9)
Very low	45 (25.6)	17 (9.7)	117 (66.5)	115 (65.3)
No answer	7 (4.0)	6 (3.4)	10 (5.7)	10 (5.7)

**Table 5:** Preferred collection method among the 176 women who returned their questionnaires.

Age category (years)	No. women	Preferred clinician-collectio*n* (%)	Preferred self-collection (%)	No preference (%)	No answer (%)
30–39	86	43 (50.0)	37 (43.0)	3 (3.5)	3 (3.5)
40–49	56	30 (53.6)	19 (33.9)	5 (8.9)	2 (3.6)
≥50	34	17 (50.0)	16 (47.1)	0 (0.0)	1 (2.9)
Total	176	90 (51.1)	72 (40.9)	8 (4.6)	6 (3.4)

## 4 Discussion

The incidence and mortality rate of cervical cancer remain high in Poland. To improve the early detection of precancerous lesions and cervical cancers, a new scheme, which involves performing an hr-HPV test with limited genotyping, was implemented in the Polish cervical cancer screening program (15). A shift toward molecular testing could facilitate the introduction of additional sampling methods, such as vaginal self-sampling, which could help improve the currently low screening coverage of the publicly funded Polish program (18, 19). Numerous studies have evaluated the agreement between self-collected and clinician-collected samples for HPV testing, as well as women's acceptance of self-collection, demonstrating that self-sampling is both accurate and highly acceptable (21). However, to our knowledge, this is the first study to investigate the concordance of HPV detection between self-collected vaginal samples and clinician-collected cervical samples in Poland.

Importantly, the present study was conducted between December 2015 and November 2018, i.e., before the nationwide implementation of HPV-based cervical screening in Poland. Therefore, the observed participation patterns and attitudes toward self-sampling reflect the pre-implementation setting. Population awareness, acceptance and long-term real-world data from the post-implementation period are still limited.

In this study, vaginal self-sampling using the Evalyn^®^ Brush was well accepted. Most women (>82%) found it convenient, comfortable and easy to perform (Table 3). All 180 participants provided suitable samples for molecular analysis, as indicated by the successful amplification of the internal control in every case. Clinician-collected samples were also adequate, resulting in comparable HPV positivity rates (65.6% for self-sampling and 66.7% for clinician-sampling).

The HPV prevalence observed here was higher than that reported for women in the general Polish population (16.6%) (22), but consistent with prevalence rates found in women with abnormal cytology or those from screening cohorts (range 47.2%−70.1%) (23–25). The high positivity rates in our study are likely attributable to the inclusion of women with previously unclear or abnormal cytology results. Thus, the prevalence observed in our cohort should be interpreted in the context of a higher-risk population rather than the general screening population.

We observed a substantial overall agreement (88.9%, κ = 0.75) between self-collected vaginal and clinician-collected cervical samples. Similar concordance (88.7%, κ = 0.72) was reported in a large meta-analysis comparing self- and clinician-collected samples for HPV testing in cervical cancer screening (26). The high agreement supports the clinical validity of self-sampling as a potential addition to national screening strategies.

Self-sampling has been shown to increase participation among women who otherwise avoid screening. In Finland, attendance among previous non-attendees increased by 17% when self-sampling was offered as a follow-up option (27). Similarly, the introduction of HPV self-sampling in Sweden during the COVID-19 pandemic increased the national screening coverage from 54%−60% across all age groups within 1 year (28). The age range of participants in our study (30–70 years) covered the target of the Polish program (25–64 years). Although HPV prevalence varied slightly between age categories, concordance between self-collected and clinician-collected samples did not differ substantially (Table 2). This indicates that self-sampling is suitable for all women eligible for screening in Poland. Moreover, since approximately one fourth of the new cervical cancer cases in Poland occur in women over 60 years of age (29), self-sampling could also serve as a valuable tool for extending screening access to older women, who may find clinician-based sampling uncomfortable or inconvenient.

High-risk HPV types 16 and 18 are responsible for a majority of cervical cancer cases. In Poland, these two types together account for approximately 88% of all cervical cancers (30). In our cohort, 18.3% and 4.4% of the self-collected samples were positive for HPV 16 and 18, respectively, compared to 18.9% and 6.7% of the clinician-collected samples. The agreement for detection of HPV 16 and HPV 18 was 96.1% (κ = 0.87) and 94.4% (κ = 0.47), respectively, indicating very good concordance for HPV 16 and moderate agreement for HPV 18.

A prerequisite for achieving self-sampling accuracies comparable to clinically collected samples is the use of validated hr-HPV assays based on PCR (31). The EUROArray HPV test used in this study is such a clinically validated, CE-marked diagnostic system, capable of simultaneously detecting and differentiating 30 anogenital HPV types, including 18 hr-HPV types (32, 33). Previous comparative evaluations have demonstrated that the EUROArray HPV performs equivalently to other commercial assays, such as the Hybrid Capture 2 (HC2) and Cobas 4800 HPV, for detecting clinically relevant high-risk genotypes (34). This reinforces the confidence that the agreement observed here reflects true analytical equivalence rather than methodological bias. All but three types (HPV 11, HPV 35, and HPV 72) were detected in the cohort. HPV 16 and HPV 42 were the most prevalent types in clinician-collected and self-collected samples, respectively (Figure 1). Extended HPV genotyping not only provides valuable information for risk determination in cervical cancer screening but also plays a role in monitoring the effectiveness of HPV vaccination programs (35–37). However, HPV vaccination status was unknown in our cohort and may have influenced the observed HPV prevalence and genotype distribution. Future studies should incorporate vaccination status in order to better assess its potential impact on HPV detection pattern.

Agreement on genotype detection between the two sampling methods exceeded 95% for 22 of the 27 detected genotypes (Table 1). However, certain HPV types showed differences in detection frequency: hr-HPV type 26 (*p* = 0.037) and lr-HPV type 40 (*p* = 0.026) were more commonly detected in clinician-collected samples, while lr-HPV type 42 was more frequent in self-collected samples. These differences likely reflect variations in tissue tropism: hr-HPV types preferentially infect the cervical transformation zone, whereas lr-HPV are more often found in the vaginal epithelium (38–43).

Multiple-type infections were slightly more common in self-collected samples (58.5%) than in clinician-collected samples (51.7%), though the difference was not statistically significant. Previous studies have reported that multiple hr-HPV infections may prolong infection duration and increase the risk of high-grade squamous intraepithelial lesions (HSIL) and cervical cancer, although the results are not conclusive (44–46). Infection multiplicity is not currently considered in triage Study limitations, however, extended genotyping is recommended to monitor infection dynamics and to distinguish between new and persistent infections (47).

In addition to screening, primary prevention through HPV vaccination is becoming increasingly relevant in Poland. The implementation of HPV vaccination within the Polish National Immunization Program represents an important opportunity to reduce the long-term burden of cervical cancer. Its strengths include national coordination, public funding, and the possibility of integrating vaccination with education on HPV-related disease prevention. At the same time, several challenges remain, including historically low vaccination uptake, hesitancy, misinformation, socioeconomic disparities, and organizational barriers. Previous studies from Poland have shown that both patient awareness and physician knowledge may affect participation in the HPV prevention strategies, underscoring the importance of effective communication and education (48, 49).

### 4.1 Study limitation

This study has several limitations. The relatively small sample size limits the generalizability of the findings. Recruitment from clinical settings might have introduced selection bias, as participants all had a history of abnormal cytology and may not represent the general screening population. Furthermore, the lack of clinical outcome data (cytology or histology) prevents conclusions about the comparative clinical accuracy (i.e., CIN2+ detection) of self- vs. clinician-collected samples. In addition, socioeconomic status and ethnicity were not systematically collected and therefore could not be included in the present analysis. Data on sexual history and HPV vaccination status were also not available. These factors may influence HPV prevalence, genotype distribution, and participation in screening and should be considered in future studies. Lastly, while most women found self-sampling easy and acceptable, slightly more than half preferred clinician-collected sampling, which might reflect limited awareness or confidence in self-collection among Polish women. Educational campaigns and clear guidance could help improve future acceptance.

### 4.2 Conclusion

This study provides valuable new data on the feasibility and reliability of vaginal self-sampling for HPV detection in Poland. Vaginal self-sampling using the Evalyn^®^ Brush demonstrated substantial agreement with clinician-collected cervical samples when analyzed with the EUROArray HPV test. Self-sampling was well-accepted by participants and could serve as a practical and effective alternative to clinician-based sampling in the Polish cervical cancer screening program. Integrating self-sampling into the national program could not only increase participation but also reach women in rural or underserved areas where gynecological care is limited. By combining molecular HPV testing with self-sampling, Poland could significantly improve screening coverage, facilitate early detection, and ultimately reduce cervical cancer incidence and mortality.

## Data Availability

The raw data supporting the conclusions of this article will be made available by the authors, without undue reservation.

## References

[1] International WCRF. Cervical cancer statistics (2022). Available online at: https://www.wcrf.org/preventing-cancer/cancer-statistics/cervical-cancer-statistics/ (Accessed April 8, 2026).

[2] Bruni L Albero G Serrano B Mena M Collado JJ Gómez D . Human Papillomavirus and Related Diseases in the World. Summary Report. Barcelona: ICO/IARC Information Centre on HPV and Cancer (HPV Information Centre), L'Hospitalet de Llobregat (2023).

[3] Kashyap N Krishnan N Kaur S Ghai S. Risk factors of cervical cancer: a case-control study. Asia Pac J Oncol Nurs. (2019) 6:308–14. doi: 10.4103/apjon.apjon_73_18 31259228 PMC6518992

[4] Oscarsson MG Benzein EG Wijma BE. Reasons for non-attendance at cervical screening as reported by non-attendees in Sweden. J Psychosom Obstet Gynecol. (2008) 29:23–31. doi: 10.1080/01674820701504619 18266164

[5] Waller J Bartoszek M Marlow L Wardle J. Barriers to cervical cancer screening attendance in England: a population-based survey. J Med Screen. (2009) 16:199–204. doi: 10.1258/jms.2009.009073 20054095

[6] Gizaw M Teka B Ruddies F Kassahun K Worku D Worku A . Reasons for not attending cervical cancer screening and associated factors in rural Ethiopia. Cancer Prev Res. (2020) 13:593–600. doi: 10.1158/1940-6207.CAPR-19-0485 32371553

[7] Tranberg M Bech BH Blaakær J Jensen JS Svanholm H Andersen B. Study protocol of the CHOiCE trial: a three-armed, randomized, controlled trial of home-based HPV self-sampling for non-participants in an organized cervical cancer screening program. BMC Cancer. (2016) 16:835. doi: 10.1186/s12885-016-2859-z 27809810 PMC5094020

[8] Ronco G Dillner J Elfström KM Tunesi S Snijders PJ Arbyn M . Efficacy of HPV-based screening for prevention of invasive cervical cancer: follow-up of four European randomised controlled trials. Lancet. (2014) 383:524–32. doi: 10.1016/S0140-6736(13)62218-7 24192252

[9] Arbyn M Verdoodt F Snijders PJ Verhoef VM Suonio E Dillner L . Accuracy of human papillomavirus testing on self-collected versus clinician-collected samples: a meta-analysis. Lancet Oncol. (2014) 15:172–83. doi: 10.1016/S1470-2045(13)70570-9 24433684

[10] Chao Y-S Clark M Ford C. HPV Self-Sampling for Primary Cervical Cancer Screening: A Review of Diagnostic Test Accuracy and Clinical Evidence. Ottawa: Canadian Agency for Drugs and Technologies in Health: (CADTH Rapid response report: summary with critical appraisal) (2018). 30329248

[11] Gupta S Palmer C Bik EM Cardenas JP Nuñez H Kraal L . Self-sampling for human papillomavirus testing: increased cervical cancer screening participation and incorporation in international screening programs. Front Public Health. (2018) 6:77. doi: 10.3389/fpubh.2018.00077 29686981 PMC5900042

[12] Nowakowski A Cybulski M Sliwczyński A Chil A Teter Z Seroczyński P . The implementation of an organised cervical screening programme in Poland: an analysis of the adherence to European guidelines. BMC Cancer. (2015) 15:279. doi: 10.1186/s12885-015-1242-9 25879466 PMC4417537

[13] Nowakowski A. Cervical cancer - a preventable (?) disease in Poland. Ginekol Pol. (2023) 94:947–9. doi: 10.5603/gpl.98540 38099662

[14] Januszek-Michalecka L Nowak-Markwitz E Banach P Spaczynski M. Effectiveness of the National Population-Based Cervical Cancer Screening Programme in Poland – Outcomes, problems and possible solutions 7 years after implementation. Ann Agric Environ Med. (2013) 20:859–64. 24364469

[15] Glinska P Komerska K Janik B Olkowicz J Jedrzejewska I Macios A . HPV testing in Polish population-based cervical cancer screening programme (HIPPO project)-study protocol of a randomised healthcare policy trial. BMC Cancer. (2023) 23:1118. doi: 10.1186/s12885-023-11597-5 37978452 PMC10655392

[16] Komerska K Macios A Glińska P Olszewski W Didkowska J Wojciechowska U . Why are Polish women diagnosed with invasive cervical cancer after negative cytology in the organized screening programme - a pilot reevaluation of negative Pap smears preceding diagnoses of interval cancers. Pol J Pathol. (2021) 72:261–6. doi: 10.5114/pjp.2021.112832 35048639

[17] Sniadecki M Poniewierza P Jaworek P Szymańczyk A Andersson G Stasiak M . Thousands of women's lives depend on the improvement of Poland's cervical cancer screening and prevention education as well as better networking strategies amongst cervical cancer facilities. Diagnostics. (2022) 12:1807. doi: 10.3390/diagnostics12081807 35892517 PMC9394414

[18] Seweryn M Leszczyńska A Jakubowicz J Banaś T. Cervical cancer in Poland — epidemiology, prevention, and treatment pathways. Oncol Clin Pract. (2025) 21:391–8. doi: 10.5603/ocp.100857

[19] Januszewski M Ziuzia-Januszewska LM Durczynski A Holubka J Siwek B Siwek Z . Traditional vs novel out-of-office method for collecting cytology and HPV DNA - a comparative study. Ginekol Pol. (2025) 96:81–6. doi: 10.5603/gpl.100294 39287210

[20] Landis JR Koch GG. The measurement of observer agreement for categorical data. Biometrics. (1977) 33:159–74. doi: 10.2307/2529310 843571

[21] Nelson EJ Maynard BR Loux T Fatla J Gordon R Arnold LD. The acceptability of self-sampled screening for HPV DNA: a systematic review and meta-analysis. Sex Transm Infect. (2017) 93:56. doi: 10.1136/sextrans-2016-052609 28100761

[22] Bardin A Vaccarella S Clifford GM Lissowska J Rekosz M Bobkiewicz P . Human papillomavirus infection in women with and without cervical cancer in Warsaw, Poland. Eur J Cancer. (2008) 44:557–64. doi: 10.1016/j.ejca.2007.12.001 18191395

[23] Bebyn MG Sledzińska P Wojtysiak J Józwicki W Mierzwa T Dziedzic J . HPV RNA and DNA testing in Polish women screened for cervical cancer – A single oncological center study. Eur J Obstet Gynecol Reprod Biol. (2022) 268:129–34. doi: 10.1016/j.ejogrb.2021.11.427 34915392

[24] Przybylski M Pruski D Wszołek K de Mezer M Żurawski J Jach R . Prevalence of HPV and assessing type-specific HPV testing in cervical high-grade squamous intraepithelial lesions in Poland. Pathogens. (2023) 12:350. doi: 10.3390/pathogens12020350 36839622 PMC9963087

[25] Glinska P Macios A Jaworski R Bobinski M Pruski D Przybylski M . Baseline data on distribution of human papillomavirus (HPV) genotypes in cervical samples of gynecological patients before implementation of population-based HPV vaccination program in Poland. Ginekol Pol. (2024) 95:870–8. doi: 10.5603/gpl.101436 39287203

[26] Arbyn M Castle PE Schiffman M Wentzensen N Heckman-Stoddard B Sahasrabuddhe VV. Meta-analysis of agreement/concordance statistics in studies comparing self- vs clinician-collected samples for HPV testing in cervical cancer screening. Int J Cancer. (2022) 151:308–12. doi: 10.1002/ijc.33967 35179777

[27] Virtanen A Nieminen P Luostarinen T Anttila A. Self-sample HPV tests as an intervention for nonattendees of cervical cancer screening in Finland: a randomized trial. Cancer Epidemiol Biomarkers Prev. (2011) 20:1960–9. doi: 10.1158/1055-9965.EPI-11-0307 21752985

[28] Elfström M Gray PG Dillner J. Cervical cancer screening improvements with self-sampling during the COVID-19 pandemic. Elife. (2023) 12:e80905. doi: 10.7554/eLife.80905 38085566 PMC10715724

[29] Nowakowski A Cybulski M Buda I Janosz I Olszak-Wasik K Bodzek P . Cervical cancer histology, staging and survival before and after implementation of organised cervical screening programme in Poland. PLoS ONE. (2016) 11:e0155849. doi: 10.1371/journal.pone.0155849 27196050 PMC4873170

[30] Cancer IIICoHa. Poland: Human Papillomavirus and Related Cancers, Fact Sheet. Barcelona: ICO/IARC Information Centre on HPV and Cancer (HPV Information Centre), L'Hospitalet de Llobregat (2023).

[31] Arbyn M Smith SB Temin S Sultana F Castle P Collaboration Collaboration on Self-Sampling and HPV Testing. Detecting cervical precancer and reaching underscreened women by using HPV testing on self samples: updated meta-analyses. BMJ. (2018) 363:k4823. doi: 10.1136/bmj.k4823 30518635 PMC6278587

[32] Viti J Poljak M Oštrbenk A Bhatia R Alcañiz Boada E Cornall AM . Validation of EUROArray HPV test using the VALGENT framework. J Clin Virol. (2018) 108:38–42. doi: 10.1016/j.jcv.2018.09.005 30223253

[33] van den Borst E Vanden BD De SP Donders G Doyen J Tjalma W . EUROArray HPV test accuracy for cervical precancer in self- vs. clinician-collected samples using the VALHUDES protocol. Gynecol Oncol. (2025) 202:14–23. doi: 10.1016/j.ygyno.2025.08.018 40934859

[34] Cornall AM Poljak M Garland SM Phillips S Machalek DA Tan JH . EUROarray human papillomavirus (HPV) assay is highly concordant with other commercial assays for detection of high-risk HPV genotypes in women with high grade cervical abnormalities. Eur J Clin Microbiol Infect Dis. (2016) 35:1033–6. doi: 10.1007/s10096-016-2634-8 27048314

[35] Bonde JH Sandri MT Gary DS Andrews JC. Clinical utility of human papillomavirus genotyping in cervical cancer screening: a systematic review. J Low Genit Tract Dis. (2020) 24:1–13. doi: 10.1097/LGT.0000000000000494 31714325 PMC6924950

[36] Demarco M Hyun N Carter-Pokras O Raine-Bennett TR Cheung L Chen X . A study of type-specific HPV natural history and implications for contemporary cervical cancer screening programs. EClinicalMedicine. (2020) 22:100293. doi: 10.1016/j.eclinm.2020.100293 32510043 PMC7264956

[37] Loenenbach A Schönfeld V Takla A Wiese-Posselt M Marquis A Thies S . Human papillomavirus prevalence and vaccine effectiveness in young women in Germany, 2017/2018: results from a nationwide study. Front Public Health. (2023) 11:1204101. doi: 10.3389/fpubh.2023.1204101 37719724 PMC10501861

[38] Gravitt PE Lacey Jr JV Brinton LA WA Kornegay JR Greenberg MD . Evaluation of self-collected cervicovaginal cell samples for human papillomavirus testing by polymerase chain reaction. Cancer Epidemiol Biomarker Prev. (2001) 10:95–100. 11219778

[39] Gravitt PE Belinson JL Salmeron J Shah KV. Looking ahead: a case for human papillomavirus testing of self-sampled vaginal specimens as a cervical cancer screening strategy. Int J Cancer. (2011) 129:517–27. doi: 10.1002/ijc.25974 21384341 PMC3782104

[40] Jones HE Allan BR van de Wijgert JH Altini L Taylor SM de Kock A . Agreement between self- and clinician-collected specimen results for detection and typing of high-risk human papillomavirus in specimens from women in Gugulethu, South Africa. J Clin Microbiol. (2007) 45:1679–83. doi: 10.1128/JCM.02369-06 17409209 PMC1933028

[41] Petignat P Faltin DL Bruchim I Tramèr MR Franco EL Coutlée F. Are self-collected samples comparable to physician-collected cervical specimens for human papillomavirus DNA testing? A systematic review and meta-analysis. Gynecol Oncol. (2007) 105:530–5. doi: 10.1016/j.ygyno.2007.01.023 17335880

[42] Castle PE Rodriguez AC Porras C Herrero R Schiffman M Gonzalez P . A comparison of cervical and vaginal human papillomavirus. Sex Transm Dis. (2007) 34:849–55. doi: 10.1097/OLQ.0b013e318064c8c5 17621246 PMC3962831

[43] Ketelaars PJW Bosgraaf RP Siebers AG Massuger LFAG van der Linden JC Wauters CAP . High-risk human papillomavirus detection in self-sampling compared to physician-taken smear in a responder population of the Dutch cervical screening: results of the VERA study. Prev Med. (2017) 101:96–101. doi: 10.1016/j.ypmed.2017.05.021 28579497

[44] Walboomers JM Jacobs MV Manos MM Bosch FX Kummer JA Shah KV . Human papillomavirus is a necessary cause of invasive cervical cancer worldwide. J Pathol. (1999) 189:12–19. doi: 10.1002/(SICI)1096-9896(199909)189:1<12::AID-PATH431>3.0.CO;2-F 10451482

[45] Campos NG Rodriguez AC Castle PE Herrero R Hildesheim A Katki H . Persistence of concurrent infections with multiple human papillomavirus types: a population-based cohort study. J Infect Dis. (2011) 203:823–7. doi: 10.1093/infdis/jiq131 21257737 PMC3071138

[46] Na J Li Y Wang J Wang X Lu J Han S. The correlation between multiple HPV infections and the occurrence, development, and prognosis of cervical cancer. Front Microbiol. (2023) 14:1220522. doi: 10.3389/fmicb.2023.1220522 37577444 PMC10416435

[47] Massad LS Clarke MA Perkins RB Garcia F Chelmow D Cheung LC . Applying results of extended genotyping to management of positive cervicovaginal human papillomavirus test results: enduring guidelines. J Low Genit Tract Dis. (2025) 29:134–43. doi: 10.1097/LGT.0000000000000865 39791481 PMC11939109

[48] Trojnarska D Baran R Babczyk D Jach R. A survey of knowledge, attitudes and awareness of the HPV and HPV vaccine among obstetricians and gynecologists across Poland. Ginekol Pol. (2022) 93:872–80. doi: 10.5603/GP.a2021.0228 35072233

[49] Trojnarska D Jach R. Primary prevention of HPV-related diseases from the patients' perspective in Poland. Eur J Cancer Prev. (2024) 33:299–308. doi: 10.1097/CEJ.0000000000000866 38113130 PMC11155277

[50] Ministry of Health. Profilaktyka raka szyjki macicy (2019). Available online at: https://pacjent.gov.pl/program-profilaktyczny/profilaktyka-raka-szyjki-macicy (Accessed April 8, 2026).

[51] R Core Team. R: A Language and Environment for Statistical Computing. Vienna, Austria: R Foundation for Statistical Computing (2019). Available online at: https://www.R-project.org/ (Accessed April 8, 2026).

